# Exhale-Focused Thermal Image Segmentation Using Optical Flow-Based Frame Filtering and Transformer-Aided Deep Networks

**DOI:** 10.3390/bioengineering12050542

**Published:** 2025-05-18

**Authors:** Do-Kyeong Lee, Jae-Sung Shin, Jae-Sung Choi, Min-Hyung Choi, Min Hong

**Affiliations:** 1 Department of Software Convergence, Soonchunhyang University, Asan 31538, Republic of Korea; dooky606@daum.net (D.-K.L.); newcastle1031@gmail.com (J.-S.S.); 2 Department of Internal Medicine, Cheonan Hospital, College of Medicine, Soonchunhyang University, Cheonan 31151, Republic of Korea; cjssch@schmc.ac.kr; 3 Department of Computer Science, Saint Louis University, Louis, MO 63103, USA; min.choi@slu.edu; 4 Department of Computer Software Engineering, Soonchunhyang University, Asan 31538, Republic of Korea

**Keywords:** thermal imaging, non-contact pulmonary monitoring, U-Net-based segmentation, pulmonary diagnostics

## Abstract

Since the COVID-19 pandemic, interest in non-contact diagnostic technologies has grown, leading to increased research into remote biosignal monitoring. The respiratory rate, widely used in previous studies, offers limited insight into pulmonary volume. To redress this, we propose a thermal imaging-based framework for respiratory segmentation aimed at estimating non-invasive pulmonary function. The proposed method uses an optical flow magnitude-based thresholding technique to automatically extract exhalation frames and segment them into frame sequences. A TransUNet-based network, combining a Convolutional Neural Network (CNN) encoder–decoder architecture with a Transformer module in the bottleneck, is trained on these sequences. The model’s Accuracy, Precision, Recall, IoU, Dice, and F1-score were 0.9832, 0.9833, 0.9830, 0.9651, 0.9822, and 0.9831, respectively, which results demonstrate high segmentation performance. The method enables the respiratory volume to be estimated by detecting exhalation behavior, suggesting its potential as a non-contact tool to monitor pulmonary function and estimate lung volume. Furthermore, research on thermal imaging-based respiratory volume analysis remains limited. This study expands upon conventional respiratory rate-based approaches to provide a new direction for respiratory analysis using vision-based techniques.

## 1. Introduction

Respiratory diseases, including pneumonia, chronic obstructive pulmonary disease (COPD), and post-COVID-19 complications, have emerged as significant global health issues. The prevalence of chronic pulmonary conditions continues to rise due to factors like smoking, environmental changes resulting from urbanization, and the accelerating pace of population aging [1,2]. Early diagnosis and continuous monitoring of respiratory diseases are essential to maintain pulmonary function [3]. However, conventional pulmonary function tests (PFTs) are primarily contact-based and require frequent hospital visits, which may pose limitations in terms of economic and spatial accessibility—especially for the elderly and individuals vulnerable to infections [4,5].

In particular, infectious respiratory diseases like COVID-19 increase the burden on healthcare facilities from the need for strict hygiene management to prevent the spread of infection. Furthermore, the currently widespread pulmonary function test (PFT) [6] requires repeated forced respiration through a mouthpiece, which may cause patient discomfort and lead to variability in test results, depending on the individual’s level of effort [7]. In consequence, there has been growing interest in the development of non-contact pulmonary function assessment technologies [8,9,10,11,12,13], with image- and signal-based respiratory analysis techniques attracting increasing attention [14,15,16,17].

Non-contact biosignal measurement technologies aim to transform traditionally contact-based approaches into contactless forms, thereby minimizing physical interaction with the subject. As a result, such methods are considered non-invasive and allow for broader applicability in various monitoring scenarios [18,19]. In particular, image-based analysis offers the advantage of avoiding the attachment of physical sensors, which is especially beneficial for subjects with sensitive skin (e.g., newborns), as it eliminates the risk of skin irritation or detachment while enabling diverse physiological assessments.

Previous studies of image- or video-based respiratory analysis have primarily focused on respiratory rate estimation, apnea detection, and inspiration/expiration pattern classification [20,21,22]. Typically, respiratory activity is monitored by detecting either the nasal or thoracic region. In nasal analysis, temperature variations in the region of interest (ROI) are used to distinguish between inhalation and exhalation phases. In thoracic analysis, the upward and downward motion of the chest is tracked to estimate respiratory cycles. Although these approaches have demonstrated high reliability in measuring respiratory rate and detecting apnea, they face inherent limitations in quantifying respiratory volume. This limitation arises primarily from their reliance on indirect or localized signals, without incorporating critical parameters, such as spatial dispersion, flow velocity, or exhalation force.

This study proposes a deep learning-based framework that uses thermal imaging for exhalation segmentation and respiratory region estimation. Through optical flow analysis, exhalation frames are automatically extracted, and the resulting image sequences are used to train a TransUNet-based segmentation network. This enables precise segmentation of the respiratory region, allowing for more accurate quantification of the total respiratory volume. The main contributions of this study are as follows:

1. By automatically extracting exhalation and segmenting the respiratory region through thermal imaging, the proposed methodology establishes the foundation for precise, personalized analysis of individual respiratory patterns.
2. The non-contact pulmonary function assessment methodology of this study suggests the potential implementation of the proposed framework in future remote healthcare services and as an assistive diagnostic tool.

## 2. Related Works

### 2.1. Pulmonary Diagnosis and Research Through Respiration

As modern technology advances, various medical and wellness technologies for pulmonary diagnosis have become increasingly diverse and sophisticated. Currently, Pulmonary Function Tests (PFTs) and high-resolution imaging technologies based on computed tomography (CT) are widely used as fundamental approaches to diagnose pulmonary diseases [23,24,25].

PFT primarily evaluates pulmonary function based on the patient’s forced expiration effort. A representative method involves the use of a spirometer, which measures indicators such as vital capacity (VC), forced vital capacity (FVC), forced expiratory volume (FEV), expiratory reserve volume (ERV), and tidal volume (TV) [25].

Also, respiration-based monitoring technologies are being applied in various ways. For precise diagnostics that require the measurement of lung volumes (e.g., residual volume and total lung capacity), plethysmography [26] is employed, and is particularly effective at diagnosing restrictive pulmonary diseases. On the other hand, in emergency situations or critical care settings where continuous monitoring of the patient’s respiratory status is necessary, capnography [27] is used to continuously track the concentration of carbon dioxide during expiration. Although these methods allow for the accurate assessment of a patient’s condition, they are often limited by high costs and restricted accessibility.

Recent advances in both hardware and software have made it possible to measure precise biological signals from target subjects [8,9,10,11,12,13,14,15,16,17,28,29]. As a result, numerous studies have been conducted on pulmonary function assessment and the diagnosis, treatment, and monitoring of pulmonary diseases. Research related to pulmonary function and disease typically begins with studies that focus on respiratory monitoring, and is later extended to include diagnostic or clinical decision support that aims to distinguish between patients and non-patients based on differences in pulmonary function. These studies can be broadly categorized into two approaches: contact-based methods [30,31,32], such as patches and belts, and non-contact-based methods [14,15,16,17], including cameras and optical sensors.

To evaluate pulmonary function, it is fundamentally necessary to continuously measure respiratory signals over a certain period. While contact-based methods allow for the continuous acquisition of biological signals, they are prone to noise due to subject movement and changes in device placement. These methods typically infer respiratory patterns by observing thoracic motion or by identifying heat-emitting regions associated with breathing. Although such studies can estimate patterns of expiration and inspiration, they face limitations in accurately correlating these patterns with quantitative lung volumes and the actual amount of respiration. Recently, research has expanded beyond respiratory pattern analysis to include investigations into lung volume and respiratory volume [13,33]. For example, as an alternative to traditional CO_2_-based methods, a novel approach has been proposed that uses thermal imaging to track respiratory molecules and quantitatively measure respiratory volume.

### 2.2. U-Net

U-NET is a U-shaped CNN-based network structure proposed by Ronneberger et al. in 2015 [34]. This consists of a total of 23 convolutional layers, and efficiently maintains spatial information through a Contracting path (encoder)–Expanding path (decoder) structure, and Skip connections to minimize pixel loss. Each stage of the encoder reduces the spatial resolution of feature maps while increasing the number of filters through continuous pooling. The decoder, connected through Skip connections, integrates global information with local information to efficiently maintain spatial information. The most significant characteristic of U-Net is its ability to perform efficient learning with limited data, which is why it offers diverse applications in images where data are scarce [35,36,37,38].

The original U-Net architecture is currently being applied across a wide range of studies, and several extended versions—such as 3D U-Net [39], Attention U-Net [40], TransUNet [41], U-Net++ [42], and ResUNet [43]—have been developed and adopted in various domains to address more complex tasks and improve segmentation performance.

This study employed TransUNet, which incorporates a Transformer module into the U-Net architecture, to effectively segment the respiratory region. The model was trained to map both local and global spatial features of respiration, to enable precise identification of respiratory patterns.

### 2.3. Thermal Camera-Based Respiratory Tracking Study

Conventional respiratory analysis studies have typically relied on attaching sensors to the subject’s body or using thermal camera-based non-contact methods to monitor respiration. However, recent exploratory studies have attempted to use thermal imaging to analyze various respiratory patterns. Schoun et al. (2017) proposed a system that employs a thermal camera and a thermally conductive medium to analyze the heat generated during expiration in real time. This study quantitatively estimated respiratory rate, breathing strength, and the distribution of oral and nasal expiration. In the fan simulation, Fast Fourier Transform (FFT)-based analysis was performed using a fixed respiratory rate of 10 BPM, and a high level of accuracy was demonstrated with an error rate within ±0.2 [44].

Transue et al. (2023) proposed a model that estimates expiratory airflow (L/s) and volume (L) using thermal CO_2_ imaging and Horn-Schunck-based optical flow analysis. In this study, optical flow and intensity signals were extracted from thermal videos of respiration, and an LSTM-based regression model was trained to predict the expiratory volume. The results demonstrated that respiratory volume could be estimated in a non-contact manner, and when compared with conventional pulmonary function tests, the method showed high accuracy (R^2^ = 0.912) [33].

While previous studies have demonstrated the potential for quantitative analysis based on respiratory imaging, they have primarily relied on waveform-based analysis. In this study, thermal imaging was used to capture respiratory videos, and expiratory airflow was segmented based on its actual spatial distribution to directly extract and analyze the respiratory volume.

## 3. Method

The goal of image-based expiration/inspiration segmentation and expiration segmentation is to estimate lung volume and describe pulmonary function. The proposed method helps visually identify the characteristics of expiration, making it possible to estimate individual breathing patterns and respiratory volume. Unlike traditional methods that only separate expiration and inspiration using the chest or nose areas, this approach captures actual expiratory volume and reflects individual respiratory traits. The pipeline is as follows:

(a) The data are divided into image sequences of 1000 frames, and a region of interest (ROI) is selected to track CO_2_ movement during expiration.
(b) Optical flow is used to analyze small pixel changes and calculate motion, allowing the system to distinguish between expiration and inspiration and label each image accordingly.
(c) The images labeled as “expiration” are input into TransUNet to predict the respiratory region around the subject’s nose and mouth, and the model performs segmentation of the breathing area.

**Figure 1:**
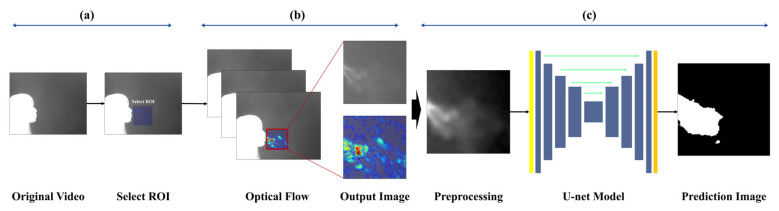
Overview of the Entire Pipeline.

Figure 1 shows an overview of this pipeline.

### 3.1. Experimental Site and Participant Exclusion Criteria

This study was approved by the Institutional Review Board (IRB) of Soonchunhyang University Cheonan Hospital (Approval No. 2023-10-012), and data were collected from Soonchunhyang University Cheonan Hospital (Cheonan, Republic of Korea) and Soonchunhyang University (Asan, Republic of Korea). Individuals who did not sign the informed consent form, expressed a desire to withdraw from the experiment, or were deemed unable to continue participation, were excluded from the study.

### 3.2. Data Collection

**Figure 2:**
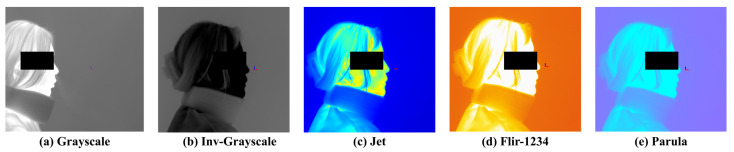
Examples of Colormaps.

Each subject participated in a single experiment lasting approximately 5 to 10 min, during which thermal video data comprising about 1000 frames (approximately 1 min in duration) was recorded using a mid-wave infrared (MWIR) thermal camera (640 × 512 pixels @ 25 Hz). Table 1 summarizes the camera specifications:

To maintain a certain level of normalization across recordings, the camera was positioned approximately 1 m distance from the participant, and captured a frontal view with the subject oriented laterally. A background screen was placed behind the participant. To minimize thermal noise, the indoor temperature was maintained at approximately 20−22 °C. The thermal camera was connected to a computer in real time, and the video was recorded by custom software developed by a collaborative research and development team. The recorded video was processed using the software’s built-in colormap adjustment tool (Dynamic Range Scaling Tool), which allowed pixel value modification. The software also enabled color correction and image preprocessing based on user settings. Figure 2 presents examples of visualization results based on different colormap selections. The available colormaps include Grayscale, Inverted Grayscale, Jet, Parula, Rainbow, FLIR-1234, Fusion, Highlight, and Cool. Among them, Grayscale and Inverted Grayscale convert the images into monochrome representations, while the remaining colormaps render the images in color using RGB values. Color-based visualizations can be further adjusted using a threshold bar to enhance the visibility of objects.

### 3.3. Data Preprocessing

The respiratory data consist of a sequence of images recorded for each frame, and appropriate image preprocessing has a significant impact on model training outcomes. Respiratory images often lack clear boundaries, and the thermal distribution in the background can vary considerably depending on the ambient room temperature and the subject’s body temperature. As well, various noise caused by non-expiratory airflow makes it difficult to establish a reliable criterion to extract valid respiratory data. In this study, particular emphasis was placed on clearly identifying and visualizing the respiratory region within the input images to enable accurate quantification of respiration.

To collect respiration-focused data, a region of interest (ROI) was defined to limit the analysis area. The ROI was set based on regions where respiratory activity was clearly observed, and was individually assigned for each subject to account for physiological differences. The ROI was fixed at 160 × 160 pixels, and expiration and inspiration behaviors were tracked within this area. To track respiratory motion, the Horn-Schunck [13,45] optical flow algorithm used in a previous study was employed. This algorithm performs parallel computation of optical flow based on the flow field mapped within the ROI, and visualizes the gradients of the resulting vectors for further analysis.

This algorithm is based on the brightness constancy assumption and the smoothness constraint of the flow field. These assumptions imply that: (1) the brightness of a pixel remains constant over time, and (2) the motion of neighboring pixels is similar.

Equation (1) gives the equation derived from the brightness constancy assumption:

(1) $$I\left(x+u,y+v,t+1\right)\approx i\left(x,y,t\right)+I_{x}u+I_{y}v+I_{t}=0$$

where $I_{x}$, $I_{y}$, and $I_{t}$ represent the partial derivatives of image intensity with respect to the *x*, *y*, and time directions, respectively, while u and v denote the pixel velocities in the *x* and *y* directions.

Equation (2) gives the equation based on the similarity of neighboring pixels:

(2) $$E_{smooth}=\iint \left[{{(\nabla }_{u})}^{2}+{{(\nabla }_{v})}^{2}\right]dxdy$$

Equation (3) presents the final energy function of the algorithm:

(3) $$E=\iint [{\left(I_{x}u+I_{y}v+I_{t}\right)}^{2}+\alpha^{2}({{|\nabla }_{u}|}^{2}+{{|\nabla }_{v}|}^{2})]dxdy$$

**Figure 3:**
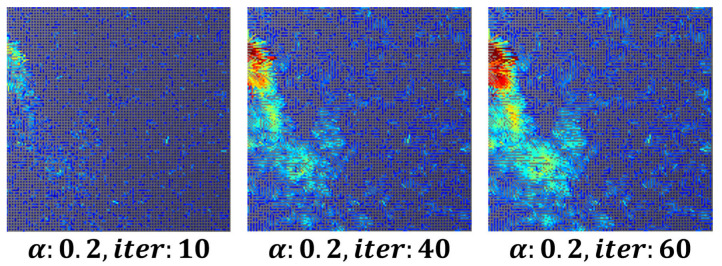
Respiration Visualization According to Parameter Changes.

To remove minor fluctuations in the flow field and achieve stable visualization, the parameter was set to α= 0.3, 80 iterations, while the number of iterations for both $u_{i}$ and $v_{i}$ increased. This approach ensured the stability of respiration visualization using image derivatives in the x and y directions. Figure 3 shows an example of a visualized image generated using the optical flow-based flow field method.

**Figure 4:**
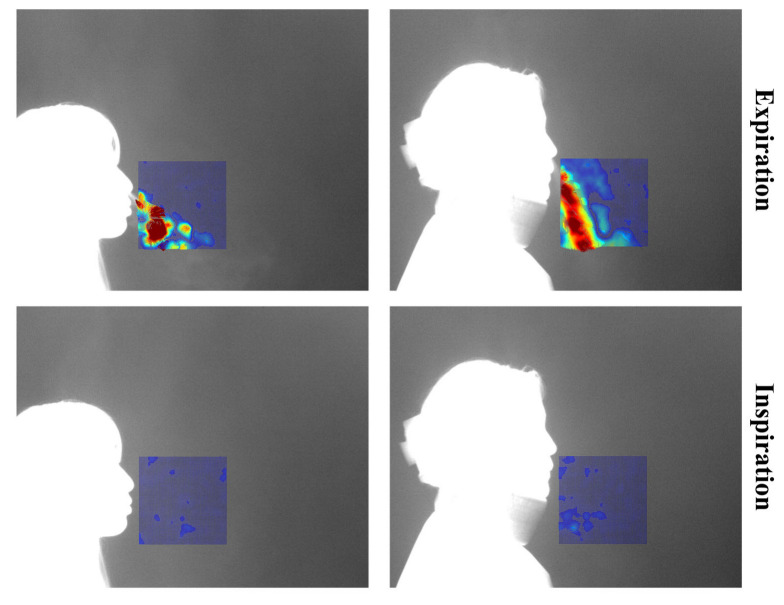
Visualization of Inspiration and Expiration Classification.

Each video consists of the first frame used to define the region of interest (ROI), followed by 999 subsequent frames for optical flow computation. These 999 frames visualize the respiratory patterns during inspiration and expiration over time. In this study, a threshold-based method was applied to distinguish between expiration and inspiration. However, classification based solely on vector flow fields can lead to errors in visually noisy environments. To address this, the proposed method excludes vectors with low directionality or indistinct flow, and determines expiration or inspiration based on the visualization of vectors with strong, consistent direction. The colors used in vector visualization are determined by the gradient magnitude of each vector: strong gradients are shown in red hues, while weak gradients appear in blue hues. Based on this, blue pixels below the threshold were removed to clarify the respiratory region. If the number of remaining (non-removed) pixels exceeded 3% of the total ROI pixels, the frame was classified as expiration; otherwise, it was classified as inspiration. Figure 4 shows the flow field visualizations for inspiration and expiration:

Images classified as “expiration” within the ROI serve as the foundation to quantify and analyze expiratory volume [13]. These images are labeled as ground truth through a four-step process.

**Figure 5:**
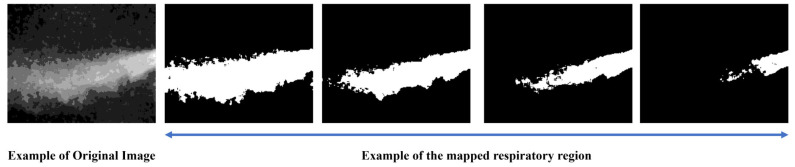
Example of Respiratory Region Definition.

Respiration can be broadly divided into three spatial regions: the core expiratory region, the surrounding region, and the background region. However, these categories can also be further subdivided into more detailed segments. Figure 5 visually illustrates the areas associated with respiration:

To generate ground truth labels, we conducted a set of experiments aimed at clearly defining the expiratory region for image preprocessing. The key considerations in the experiment were as follows:

- Definition of the core respiratory region
- Determination of the broader expiratory area
- A method for non-subjective respiratory mapping

**Figure 6:**
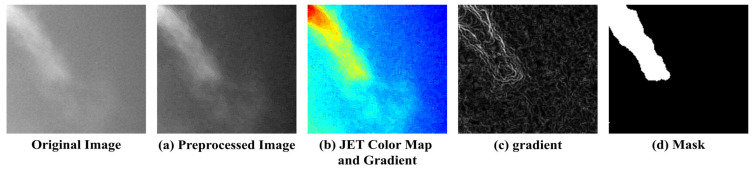
Example of Respiration Labeling.

The collected expiration images included various respiratory patterns and noise from multiple subjects, and due to individual differences, such as temperature and airflow, certain respiratory regions could be either overemphasized or overlooked. To address this, several criteria were established to consistently identify the respiratory region. Figure 6 illustrates the process of mapping the respiratory region and generating binary masks based on these criteria. (a) To reduce noise, a 5 × 5 median filter was applied, and sharpening was performed to emphasize the boundaries of the respiratory region. Subsequently, normalization was applied to smooth out pixel intensity variations, reduce noise, and enhance the visibility of the respiratory area. (b) A JET colormap was applied to separate the respiratory region by assigning consistent color values based on image brightness. (c) A gradient overlay was then performed on the color-mapped image to determine a threshold at an appropriate point where the gradient gradually became more moderate. Regions within this threshold, where noticeable brightness differences occurred due to respiration, were defined as the respiratory region, while the areas where the gradient became more gradual were classified as surrounding airflow or background. (d) Finally, the confirmed respiratory region was mapped, and a 3 × 3 median filter was applied to remove remaining salt-and-pepper noise, completing the labeling process.

### 3.4. Segmentation for Quantitative Evaluation of Respiration

#### 3.4.1. Selection of the Model

Quantitative evaluation of expiration is one of the key indicators to assess lung volume and pulmonary health status. In this study, to evaluate the expiratory volume during expiration, image sequences defined as “expiration” based on optical flow analysis were fed into an encoder, which performed binary classification between the respiratory region and the background. Segmentation of the respiratory region was conducted based on this classification. Since respiration occurs within a localized area even inside the ROI, training was performed using a U−Net-based segmentation algorithm, which is optimized to capture fine-grained and spatially limited regions.

U-Net is a network architecture that is proposed to minimize the loss of spatial information in medical image analysis. While conventional CNN-based models may lose local details due to their deep hierarchical structure, U-Net preserves global information by transferring encoder features directly to the decoder through skip connections.

In this study, the TransUNet architecture was used for training, in which CNN-based structures were applied to the encoder and decoder, and a Transformer module was inserted into the central bottleneck (bridge) layer. Table 2 shows the computing specifications used to train the model:

#### 3.4.2. TransUNet Architecture

**Figure 7:**
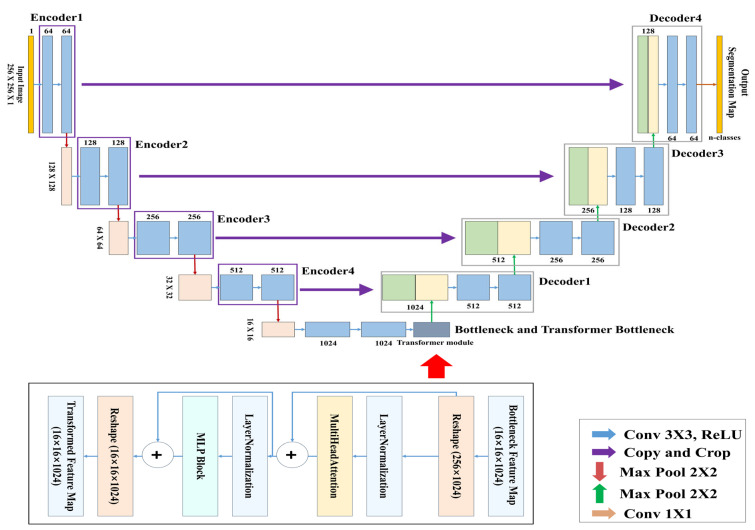
TransUNet architecture.

Figure 7 presents the complete architecture of the TransUNet used for learning and segmenting the respiratory region. This model is a segmentation network designed by combining the traditional U-Net architecture with a Transformer-based global feature encoder. The overall structure consists of three main components: the encoder, the Transformer module, and the decoder.

The objective of this study is to segment respiratory regions by training a TransUNet model to learn both the shape and global position of respiration. This is achieved through a network that predicts respiration based on its occurrence within the image and the correlation between pixels. Both the encoder and decoder are composed of standard CNN-based convolutional blocks, while the bottleneck (bridge) layer incorporates a Transformer encoder block consisting of multi-head attention and a multi-layer perceptron (MLP). Table 3 above summarizes the detailed architecture of the TransUNet used in this study.

In this study, a binary segmentation mask is generated by processing input data of size 256 × 256 × 1 through four encoder stages, followed by Transformer processing at the bottleneck, and then passing through four up-sampling decoder stages with skip connections, culminating in a sigmoid activation function at the output. While the original TransUNet typically uses ResNet-50 as the backbone encoder, this study adopts a lightweight CNN-based architecture to reduce model complexity. In addition, the global feature information extracted through the CNN encoder is tokenized at the bridge stage, processed by the Transformer module, and then passed to the decoder, allowing the model to effectively incorporate global contextual information during segmentation.

## 4. Experiments, Results, and Discussion

This section is composed of three parts: the experimental setup, the results, and the discussion. Section 4.1 describes the experimental configuration and environment for respiratory region segmentation using the lightweight TransUNet; Section 4.2 then presents the experimental results; while finally, Section 4.3 discusses the significance and limitations of the proposed method and the conducted experiments.

### 4.1. Experimental Environment

The training dataset for segmentation consists of 3645 images recorded from 10 participants. Table 4 shows the composition of the participants:

The dataset includes only frames identified as “expiration” based on the vector visualization of optical flow. Frames with discontinuity or significant noise were excluded from the dataset to ensure quality. Table 5 summarizes the experimental environment used for training the TransUNet model under various settings:

In binary classification problems, Binary Cross-Entropy (BCE) is commonly used as the loss function. However, in cases such as this study, where segmentation of localized regions (e.g., respiratory areas) is required, applying BCE—which is based on global pixel-level accuracy—may reduce the model’s ability to effectively learn small target regions. While BCE ensures overall prediction accuracy, it is limited in capturing fine structures and boundary details, which are critical when dealing with small or subtle regions.

In particular, the expiratory image data used in this study consists of a highly imbalanced class distribution, with the background (class 0) dominating over the respiratory region (class 1). To address this issue, a composite loss function was employed by combining Focal Loss, which is robust to class imbalance, with Dice Loss, which improves accuracy in capturing fine structural details. This combined loss function was applied to enhance the segmentation performance. The mathematical formulation of the composite loss function used in this study is given below. Equation (4) presents the formula for Focal Loss, while Equation (5) presents the formula for Dice Loss:

(4) $$L_{Focal}=-\frac{1}{N}\sum_{i=1}^{N}\left[\alpha \cdot y_{i}\cdot {\left(1-\hat{y_{i}}\right)}^{\gamma }\cdot \log\left(\hat{y_{i}}\right)+\left(1-\alpha \right)\cdot \left(1-y_{i}\right)\cdot \hat{y_{i}^{\gamma }}\cdot \log\left(1-\hat{y_{i}}\right)\right]$$

(5) $$L_{Dice}=1-\frac{2\sum_{i=1}^{N}y_{i}\cdot \hat{y_{i}}+\epsilon }{\sum_{i=1}^{N}y_{i}+\sum_{i=1}^{N}\hat{y_{i}}+\epsilon }$$

where $y_{i}$ denotes the ground truth mask value (either 0 or 1), $\hat{y_{i}}$ represents the predicted probability, N is the total number of pixels, and α and γ are the weighting factor for the positive class and the focusing parameter in the Focal Loss, respectively, while ϵ is a small constant added for numerical stability.

The composite loss function is calculated by summing the individual losses, such as Focal Loss and Dice Loss. In the case of weighted summation, each loss function is assigned a specific weight before being combined.

### 4.2. Experimental Results

The performance of the proposed TransUNet was evaluated using Accuracy, Precision, Recall, and F1-score, as well as segmentation-specific metrics, such as Intersection over Union (IoU), and Dice Similarity Coefficient (DSC).

The IoU and DSC evaluation metrics quantitatively assess segmentation accuracy based on the degree of overlap between the prediction and the ground truth. IoU and Dice coefficients are commonly used to measure the similarity between the segmented output and the ground truth mask. Greater overlap between the two indicates better model performance.

IoU [48] measures how much the predicted mask overlaps with the ground truth mask by dividing the area of their intersection by their area of union. The formula for IoU is given by:

(6) $$IOU=\frac{TP}{TP+FP+FN}$$

DSC [49] assigns double weight to the intersection relative to the sum of the predicted and ground truth masks. This is particularly sensitive to small objects and is widely used as an evaluation metric in segmentation tasks. The formula for DSC is given by:

(7) $$DSC=\frac{2TP}{2TP+FP+FN}$$

**Figure 8:**
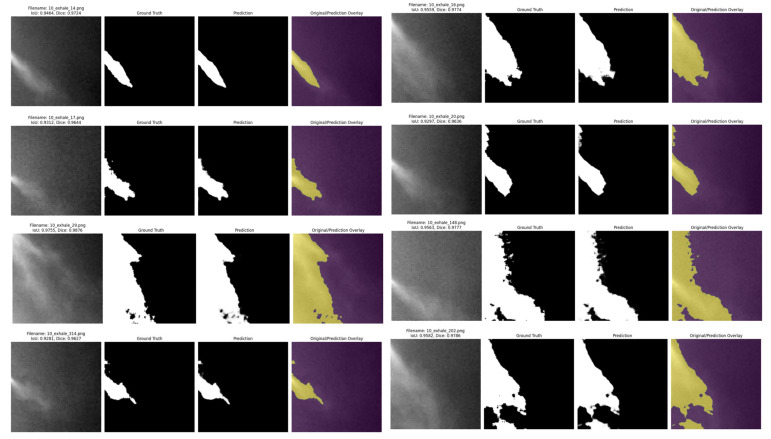
Example of TransUNet Respiratory Segmentation Visualization.

Since the respiratory region typically occupies only a small portion of the total pixel area, this study included IoU and DSC metrics to evaluate the model’s effectiveness in such localized segmentation tasks. Table 6 summarizes the results based on the evaluation metrics:

To evaluate the performance of the model, prediction testing was conducted using external data. Table 7 summarizes the results, while Figure 8 shows an example of model prediction visualization:

To evaluate the stability of the model, training was performed 10 times for 200 epochs. The results on the test data showed a mean and standard deviation of Accuracy (0.9827 ± 0.00038), IOU (0.9651 ± 0.00138), DSC (0.9822 ± 0.00072), Precision (0.9831 ± 0.00197), Recall (0.9814 ± 0.00260), and F1-score (0.9822 ± 0.00072). These results indicate that the model demonstrates excellent stability.

### 4.3. Discussion

This study performed inspiration and expiration segmentation based on CO_2_ respiratory images visualized by a thermal camera, and conducted respiratory segmentation to estimate the volume of expiration. The contributions of this study are:

- Possibility of Respiratory Pattern Analysis and Expiratory

This study attempted respiratory quantification through CO_2_ image-based segmentation without the use of additional devices. The results of this study may suggest the possibility of future respiratory quantification and pattern analysis for expiration and inspiration. The respiratory region in this study is expected to correlate with actual expiratory volume, and the results of this study may suggest the potential for the respiration and pattern analysis of inspiration/expiration to be quantified. Future follow-up research will focus on the quantification of expiration and the removal of noise to achieve more accurate segmentation of the expiratory region. As well, the dataset will be expanded to include participants of various ages, different genders, and a variety of respiratory diseases, which will facilitate the generalization and advance of segmentation for a broader range of studies.

- Potential for Non-contact Respiratory Disease Diagnosis and Clinical Support Tools

This study presents the possibility of measuring respiration without the use of wearable devices or spirometers, which could be utilized in future non-contact respiratory disease diagnosis and clinical support applications.

Therefore, it is expected that this study will be expanded into various research directions related to respiratory quantification and respiratory pattern analysis. However, there are several limitations to the current study, and additional research is needed to improve the performance. The limitations of this study are as follows:

- Limitation of segmentation due to noise

**Figure 9:**
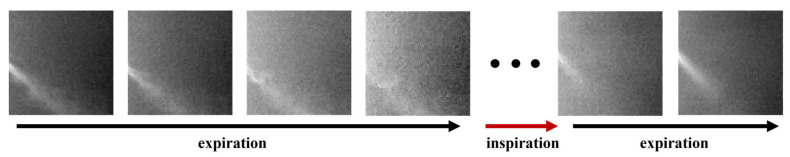
Noise generation due to changes in respiration.

In this study, respiratory patterns were classified into expiration and inspiration, and respiratory segmentation was performed when the image was classified as expiration. The respiratory image data used in this study inherently features frames that repeatedly alternate between expiration and inspiration, with residual respiration generated. The respiration begins strongly from the ${expiration\_frame}_{n}$ and starts to weaken a few frames before the onset of inspiration at the ${inspiration\_frame}_{n+1}$. Figure 9 visualizes the point at which the respiration during expiration begins to fade:

This causes noise related to respiration and leads to difficulties in accurately segmenting the expiration phase. Future research will aim to enhance model performance by applying recent self-supervised learning techniques [50] to perform more precise respiratory segmentation.

- Limitation of residual respiration segmentation

In this study, respiratory segmentation during expiration was performed. The regions where the gradient became smooth were defined as areas unrelated to direct expiration and were labeled accordingly. However, more extensive segmentation is required for accurate respiratory identification. Future research will aim to enhance segmentation performance by developing and applying algorithms that are capable of distinguishing residual respiration and noise, as part of the next phase of this study.

- Limitations of Volume Estimation Based on 2D Images

This study performed research on respiratory volume estimation based on 2D images. While image-based respiratory segmentation allows for the visualization of respiration and 2D-based estimation of respiratory volume, accurate quantification of respiratory volume requires more detailed information. In future research, studies will be conducted to quantify the respiratory volume based on the brightness of respiration.

Table 8 summarizes the contributions and limitations of this study:

## 5. Conclusions

This study performed inspiration and expiration segmentation based on thermal images and proposed a TransUNet-based model to segment the respiratory region, a localized area, using expiration image data. The segmentation of expiration and inspiration was conducted using vector visualization images based on Optical Flow, and respiratory segmentation was performed using images classified as expiration. The TransUNet model used in this study integrates a traditional CNN-based encoder–decoder structure with a Transformer module at the bottleneck to combine both local and global information, effectively capturing localized respiratory signals. To alleviate the class imbalance issue, a composite loss function combining Focal Loss and Dice Loss was applied to improve the model’s prediction performance.

The proposed model demonstrated high classification performance with an Accuracy of 0.9832, a Precision of 0.9833, a Recall of 0.9830, and an F1-score of 0.9831. In particular, it achieved a high degree of alignment with the ground truth mask, recording an IoU of 0.9669 and a DSC of 0.9831.

These results demonstrate that the proposed model effectively addresses segmentation tasks involving localized structures, such as the respiratory region, and prove its potential as a quantitative analysis tool for respiratory analysis and pulmonary function evaluation in medical imaging.

Unlike previous studies, this study attempts direct quantification of respiration by performing segmentation based on respiratory images. In contrast to earlier approaches that relied on signal waveform reconstruction, the present study proposes a novel spatial approach that enables direct extraction of the exhaled air region, thereby offering the potential to improve the precision of respiratory volume quantification.

In future research, efforts will be made to address the limitations of this study and to investigate the segmentation of various respiratory patterns. The generalization performance of the model will also be evaluated.

## Figures and Tables

**Table 1:** Specifications of the Thermal Imaging Camera.

Camera Specifications	
Model	FLIR-A6788sc (640 × 512 @ 30 Hz)
Resolution	640 × 512 pixels
Frame Rate	30–120 fps
Sensor Type	Indium Antimonide (InSb)
Spectral Range	Targeting CO_2_ absorption band in the 3–5 μm region
Camera Features	Programmatic camera control, Raw data acquisition, Embedded CO_2_ spectral filter

**Table 2:** Computing Performance.

Software/Parameters	Value
-OS	Windows 10 64-bit
Programming Language	Python (version 3.9)
CPU	Intel Core i7-7700 CPU @ 3.60 GHz
GPU	NVIDIA GeForce RTX 3090
RAM	32 GB

**Table 3:** Overview of the Entire Model.

Stage	Component	Output Size
Input		256×256×1
Encoder 1	Conv2D (64) × 2 → MaxPool	128×128×64
Encoder 2	Conv2D (128) × 2 → MaxPool	64×64×128
Encoder 3	Conv2D (256) × 2 → MaxPool	32×32×256
Encoder 4	Conv2D (512) × 2 → MaxPool	16×16×512
Bottleneck	Conv2D (1024) × 2	16×16×1024
Transformer Bottleneck	Reshape → MHA × N → MLP × N → Reshape	16×16×1024
Decoder 1	TransposeConv (512) → Concat (skip4) → Conv2D (512) × 2	32×32×512
Decoder 2	TransposeConv (256) → Concat (skip3) → Conv2D (256) × 2	64×64×256
Decoder 3	TransposeConv (128) → Concat (skip2) → Conv2D (128) × 2	128×128×128
Decoder 4	TransposeConv (64) → Concat (skip1) → Conv2D (64) × 2	256×256×64
Output	Conv2D (1 × 1) + Sigmoid	256×256×1

**Table 4:** Participant Information.

Participant Information	
Gender	5 males, 5 females (total of 10 participants)
Age	Mean: 24.3 ± 3.53 years, Range: 19−28 years
Health Status	All participants were reported to be in healthy condition based on self-assessment

**Table 5:** Experimental Environment Settings.

Experimental Setup	
Train: Validation	8: 2
Batch Size	16
Epochs	200 iterations
Learning Rate	5 × $10^{-5}$
Loss	Focal Loss + Dice Loss [46,47]
Optimizer	Adam
Performance Evaluation	Accuracy, IOU, DSC, Precision, Recall, F1-score

All values are unitless, unless specified otherwise.

**Table 6:** Model Training Results.

Performance Evaluation			
**Accuracy**	0.9832	**Precision**	0.9833
**IOU**	0.9669	**Recall**	0.9830
**DSC**	0.9831	**F1-score**	0.9831

**Table 7:** Model Test Results.

Performance Evaluation			
**Accuracy**	0.9832	**Precision**	0.9841
**IOU**	0.9665	**Recall**	0.9818
**DSC**	0.9829	**F1-score**	0.9829

**Table 8:** Contributions and Limitations of the Study.

Contributions and Limitations of This Study	
Contribution	Suggests the possibility of respiratory pattern analysis and respiratory volume estimation through quantitative respiration tracking
	Proposed potential use as an auxiliary tool for non-invasive pulmonary disease diagnosis
Limitation	Limitations in segmentation due to noise
	Limitations in segmenting subtle (low-intensity) respiration
	Limitations in volumetric estimation based on 2D images

## Data Availability

The data presented in this study are available on reasonable request from the corresponding author.
